# Comparison of IOL power calculation formulas in short eyes using ESCRS calculator and eyetemis: Influence of anterior segment biometry

**DOI:** 10.1371/journal.pone.0361501

**Published:** 2026-10-27

**Authors:** Chung Young Kim, Joo Youn Oh, Hyuk Jin Choi, Mee Kum Kim, Chang Ho Yoon

**Affiliations:** 1 Department of Ophthalmology, Seoul National University Hospital, Seoul, Republic of Korea; 2 Department of Ophthalmology, Seoul National University College of Medicine, Seoul, Republic of Korea; 3 Department of Ophthalmology, Seoul National University Hospital Healthcare System Gangnam Center, Seoul, Republic of Korea; Nicolaus Copernicus University, POLAND

## Abstract

This study compared the refractive prediction accuracy of seven contemporary intraocular lens (IOL) power calculation formulas in eyes with short axial length and assessed the influence of anterior chamber depth (ACD) and lens thickness (LT) on prediction error (PE). A total of 124 eyes with an axial length ≤22.0 mm that underwent uncomplicated phacoemulsification with implantation of a Tecnis ZCB00 IOL were included. Predicted postoperative refractions were obtained using the ESCRS calculator for Barrett Universal II, Cooke K6, EVO 2.0, Hill-RBF 3.0, Hoffer QST, Kane, and Pearl-DGS. Formula performance was evaluated using robust trimmed-mean analyses of trueness, precision, and accuracy implemented in the Eyetemis platform, along with conventional PE metrics and the proportions of eyes within predefined error thresholds. The mean axial length was 21.67 ± 0.31 mm, and the mean implanted IOL power was 25.2 ± 1.6 D. Kane showed a lower trimmed-mean absolute PE than Hoffer QST (0.376 vs. 0.445 D; p = 0.014). No significant inter-formula differences were observed at clinically relevant error thresholds. ACD and LT showed weak, formula-specific associations with PE; however, these findings were exploratory and not clinically actionable. Overall, contemporary formulas provided broadly comparable refractive accuracy in short eyes, although small formula-specific differences were observed.

## Introduction

Intraocular lens (IOL) power calculation has undergone continuous refinement over the past decades, evolving from early regression-based formulas to sophisticated theoretical, multivariate, and artificial intelligence–driven models. Initial regression formulas relied primarily on axial length and corneal curvature, often resulting in limited accuracy due to their empirical nature [1–4]. Third-generation formulas, including SRK/T, Holladay 1, and Hoffer Q, introduced theoretical optics and anterior chamber depth (ACD) –based predictions, improving refractive outcomes across a broader range of eyes [3,5,6]. More recent fourth- and fifth-generation formulas, such as Haigis, Holladay 2, Olsen, and Barrett Universal II (BU II), further enhanced predictive accuracy by incorporating additional biometric parameters and refined effective lens position (ELP) estimation [7–10]. Alongside these, artificial intelligence–based formulas that leverage large datasets have emerged, reflecting the trend toward individualized and precision-based IOL selection [11–14].

Despite these advancements, accurate IOL power calculation remains particularly challenging in eyes with short axial length. In such eyes, even minimal errors in biometric measurements or ELP prediction can be disproportionately magnified, frequently resulting in hyperopic refractive surprises [7]. Historically, Hoffer Q was considered the preferred formula for short eyes; however, subsequent studies have demonstrated improved performance with formulas such as Haigis and BU II, which more accurately model anterior segment anatomy and postoperative lens position [6,15–17]. Nevertheless, refractive predictability remains variable in short eyes, and no single formula has consistently demonstrated universal superiority.

The European Society of Cataract and Refractive Surgeons (ESCRS) IOL calculator provides unified access to multiple contemporary IOL calculation formulas under standardized biometric conditions and has gained widespread clinical adoption [18–20]. In parallel, the Eyetemis analysis tool has been developed as a web-based platform that enables ISO-compliant assessment of trueness, precision, and accuracy of spherical equivalent prediction errors using robust statistical methods [20,21]. Together, these tools allow for objective and reproducible comparison of modern IOL formulas beyond conventional mean absolute error (MAE)–based analyses. The combined ESCRS calculator–Eyetemis workflow provides clinically useful information by separating systematic prediction bias from dispersion and absolute error. Thus, rather than relying only on conventional MAE comparisons, this approach allows clinicians to determine whether an apparent difference in formula performance is related to directional bias, precision, or overall accuracy.

In this study, we aimed to evaluate and compare the refractive prediction performance of seven modern IOL calculation formulas available through the ESCRS calculator—BU II, Cooke K6, Emmetropia Verifying Optical (EVO) 2.0, Hill Radial Basis Function (RBF) 3.0, Hoffer Q Savini/Taroni (QST), Kane, and Pearl DGS—in eyes with an axial length of ≤22.0 mm. Unlike prior studies that primarily compared formula performance using conventional error metrics, the present study evaluated selected formulas available through the ESCRS calculator using the Eyetemis platform, which provides a standardized framework for assessing trueness, precision, and accuracy. This design allowed us to examine the performance of contemporary web-based formulas in a short-eye cohort under uniform default-calculator conditions and to explore whether anterior segment biometric parameters were associated with formula-specific prediction errors.

## Materials and methods

This retrospective study was approved by the Institutional Review Board of Seoul National University Hospital (SNUH; IRB No. 2112-132-1284) and adhered to the tenets of the Declaration of Helsinki. The requirement for informed consent was waived due to the retrospective nature of the study.

### Patients

We retrospectively reviewed ocular biometric data obtained using the IOLMaster 700 (Carl Zeiss Meditec, Jena, Germany). Eyes with an axial length ≤ 22.0 mm that underwent uneventful cataract surgery were considered for inclusion. Inclusion criteria were as follows: (1) age ≥ 18 years, (2) uneventful phacoemulsification with in-the-bag IOL implantation, and (3) implantation of a Tecnis^®^ ZCB00 IOL (Johnson & Johnson Vision, Jacksonville, FL, USA). The data were accessed for research purposes on December 1, 2025, and the authors did not have access to any information that could identify individual participants during or after data collection.

Exclusion criteria included: (1) previous or concomitant retinal, glaucoma, or corneal surgery; (2) corneal pathology identified by slit-lamp examination or topography; (3) intraoperative complications that could influence refractive outcomes, such as zonular dialysis, capsular tension ring implantation, or posterior capsular rupture; and (4) poor-quality biometric measurements flagged by exclamation marks (!) or asterisks (*) on the IOLMaster 700.

To avoid statistical inter-eye correlation, when both eyes of a patient met the inclusion criteria, one eye was randomly selected for analysis.

### Surgical procedure

All cataract surgeries were performed by experienced surgeons at SNUH using a standardized technique. A 2.75-mm temporal clear corneal incision was created, followed by continuous curvilinear capsulorrhexis, phacoemulsification of the crystalline lens, and implantation of the IOL into the capsular bag.

### Ophthalmic evaluation and measurement

All patients underwent comprehensive preoperative ophthalmic examinations according to the institutional cataract surgery protocol. Assessments included best-corrected visual acuity, slit-lamp biomicroscopy, automated kerato-refractometry (KR-8900; Topcon, Tokyo, Japan), anterior segment imaging (CASIA2; Tomey, Nagoya, Japan), specular microscopy (CellChek 20; Konan Medical, Irvine, CA, USA), dilated fundus examination, spectral-domain optical coherence tomography (Heidelberg Engineering, Heidelberg, Germany), ultra-widefield fundus photography (Optos California; Optos plc, Dunfermline, Scotland), and ocular biometry using the IOLMaster 700 under mesopic conditions before pupil dilation.

Preoperative biometric parameters extracted included axial length (AL), flat and steep keratometry (K), anterior chamber depth (ACD, measured from the corneal epithelium to the anterior lens surface), lens thickness (LT), central corneal thickness (CCT), and corneal diameter.

Postoperative manifest refraction was assessed 1 month after surgery. Spherical equivalent (SEQ) was calculated as the spherical refractive value plus one half of the cylindrical value. The 1-month endpoint was selected because it reflects the routine postoperative refractive assessment schedule at our institution after uncomplicated cataract surgery.

### IOL power calculation and prediction error

Prediction error (PE) was defined as the difference between the postoperative SEQ and the predicted SEQ calculated using each formula with the implanted IOL power. A negative PE indicated a myopic outcome, whereas a positive PE indicated a hyperopic outcome. The absolute prediction error (APE) was defined as the absolute value of PE.

The following metrics were calculated for each formula: mean prediction error (MPE), mean absolute error (MAE), median absolute error (MedAE), and the percentage of eyes within ±0.25, ± 0.50, ± 0.75, ± 1.00 and ±1.50 diopters (D) of the PE.

Predicted SEQ values were calculated using the ESCRS online IOL Calculator. Seven IOL power calculation formulas were evaluated: BU II (A-constant = 119.39), Cooke K6 (A-constant = 119.3), EVO 2.0 (A-constant = 119.3), Hill RBF 3.0 (A-constant = 119.34), Hoffer QST (pACD = 5.8), Kane (A-constant = 119.36), and Pearl DGS (A-constant = 119.35). The formulas were selected because they were available through the ESCRS calculator at the time of analysis. Traditional benchmark formulas that were not part of the primary ESCRS calculator workflow were not included in the main analysis. Default constants provided by the ESCRS calculator were used for all formulas. Institution-specific optimization for the study population, surgeons, IOL model, and measurement device was not performed. Therefore, the present analysis was designed to evaluate formula performance as implemented in the ESCRS calculator under standardized default-constant conditions, rather than to compare fully optimized formula performance.

### Additional analysis of biometric influence

To evaluate the influence of ACD and LT on PE, correlation analyses were performed between ACD, LT, and PE for each IOL formula using Pearson correlation coefficients. Correlation and median-split subgroup analyses were exploratory, and unadjusted P values are reported. For the multivariable regression analyses, Holm adjustment was applied to the 14 ACD/LT coefficients separately for each outcome. In addition, eyes were stratified into low and high ACD groups and low and high LT groups based on median values. The MAEs between subgroups were compared using Welch’s t-tests. Because dichotomizing continuous biometric variables may reduce statistical power, additional multivariable regression analyses were performed using PE and absolute PE as outcomes, with ACD, LT, AL, mean K, implanted IOL power, age, and sex as covariates.

### Statistical analysis

Statistical analyses were performed using SPSS software (version 27.0; IBM Corp., Armonk, NY, USA), GraphPad Prism (version 10.0; GraphPad Software, San Diego, CA, USA), and the Eyetemis analysis tool (version 0.9.09; https://eyetemis.com) [21, 22] The Eyetemis platform evaluates formula performance in accordance with ISO standards by quantifying trueness, precision, and accuracy using robust trimmed-mean statistical methods. Trueness was defined as the trimmed mean of the SEQ prediction error and was tested against zero. Precision was defined as the trimmed mean of the distances from the trimmed mean of SEQ-PE. Accuracy was defined as the trimmed mean of the absolute SEQ-PE. Pairwise comparisons between formulas were performed using robust t-tests with Holm adjustment for multiple comparisons. The percentages of eyes within each PE threshold (±0.25, ± 0.50, ± 0.75, and ±1.00 D) were compared using Cochran’s Q test, followed by pairwise McNemar tests where applicable. A significance level of α = 0.05 was applied. A priori power analysis was performed using 1,000 repetitions with a minimal detectable effect size of 0.10 D, a correlation size of 0.4, and a standard deviation of 0.34 D, yielding a statistical power of 0.868. As a sensitivity analysis, formula-specific MPE was subtracted from each PE to center the MPE at zero, and MPE-adjusted absolute PEs were recalculated for each formula. Formula differences were evaluated using the Friedman test, followed by pairwise Wilcoxon signed-rank tests with Holm adjustment.

## Results

A total of 124 eyes of 124 patients were included. The mean age at cataract surgery was 71.1 ± 10.0 years. Of these, 114 patients (91.9%) were female, and 69 eyes (55.6%) were right eyes. The mean implanted IOL power (Tecnis^®^ ZCB00) was 25.2 ± 1.6 D. The mean postoperative SEQ at 1 month was –0.39 ± 0.66 D. The mean axial length was 21.67 ± 0.31 mm, confirming a short-eye population. Detailed demographic and biometric characteristics are summarized in Table 1.

**Table 1:** Demographic and Biometric Characteristics of the Study Population.

Variable	Mean ± SD (range)
Eyes / Patients	124 / 124
Age, years	71.1 ± 10.0 (22.3–87.8)
Sex, female, n (%)	114 (91.9%)
Laterality, right, n (%)	69 (55.6%)
IOL power, D	25.2 ± 1.6 (22–31)
IOL model, n (%)	Tecnis® ZCB00, 124 (100%)
Postoperative SEQ, D	−0.39 ± 0.66 (−3.12–1.00)
AL, mm	21.67 ± 0.31 (20.35–21.99)
Flat K, D	45.86 ± 1.20 (42.68–48.93)
Steep K, D	46.81 ± 1.17 (43.23–49.42)
Mean K, D	46.33 ± 1.13 (43.23–49.18)
ACD, mm	2.50 ± 0.38 (1.67–3.53)
LT, mm	4.83 ± 0.43 (3.49–5.90)
CCT, µm	539.1 ± 37.6 (454.8–645.2)
Corneal diameter, mm	11.31 ± 0.41 (10.18–12.74)

ACD: anterior chamber depth (measured from epithelium to lens); AL: axial length; CCT: central corneal thickness; D: diopter; IOL: intraocular lens; K: keratometry; LT: lens thickness; SD: standard deviation; SEQ: spherical equivalent.

The MPE, MAE, and MedAE calculated using the seven IOL formulas are shown in Table 2. The MPEs for Barrett Universal II, Cooke K6, EVO 2.0, Hill RBF 3.0, Hoffer QST, Kane, and Pearl DGS were –0.11 ± 0.54, 0.29 ± 0.52, –0.06 ± 0.52, 0.05 ± 0.54, –0.27 ± 0.54, –0.11 ± 0.51, and 0.19 ± 0.51 D, respectively. Cooke K6, Hill RBF 3.0, and Pearl DGS showed a hyperopic tendency, whereas BU II, EVO 2.0, Hoffer QST, and Kane tended toward myopia.

**Table 2:** Refractive Outcomes of Intraocular Lens Power Calculation of Each Formula.

Formula	Constants, ZCB00	MPE ± SD, D	MAE ± SD, D	MedAE, D	Percentage of Eyes within PE (%)				
					≤±0.25D	≤ ± 0.50D	≤ ± 0.75D	≤ ± 1.00D	≤ ± 1.50D
Barrett Universal II	119.39, A constant	−0.11 ± 0.54	0.44 ± 0.33	0.35	38.71	63.71	84.68	93.55	100.00
Cooke K6	119.30, A constant	0.29 ± 0.52	0.48 ± 0.36	0.39	29.84	63.71	79.84	88.71	99.19
EVO 2.0	119.30, A constant	−0.06 ± 0.52	0.41 ± 0.32	0.33	36.29	66.13	83.87	94.35	100.00
Hill-RBF 3.0	119.34, A constant	0.05 ± 0.54	0.44 ± 0.32	0.38	32.26	59.68	86.29	94.35	98.39
Hoffer QST	5.8, pACD constant	−0.27 ± 0.54	0.48 ± 0.37	0.40	31.45	59.68	76.61	88.71	99.19
Kane	119.36, A constant	−0.11 ± 0.51	0.41 ± 0.32	0.33	38.71	65.32	81.45	95.16	100.00
Pearl DGS	119.35, A constant	0.19 ± 0.51	0.44 ± 0.32	0.43	33.06	58.06	82.26	94.35	100.00

D: diopter; MAE: mean absolute prediction error; MedAE: median absolute prediction error; MPE: mean prediction error; pACD: personalized anterior chamber depth; PE: prediction error.

Using the Eyetemis analysis tool, trimmed mean SEQ-PEs of BU II (p = 0.034), Cooke K6 (p < 0.001), Hoffer QST (p < 0.001), Kane (p = 0.026), and Pearl DGS (p < 0.001) were significantly different from zero, whereas those of EVO 2.0 (p = 0.159), and Hill RBF 3.0 (p = 0.310) were not (Fig 1A). In pairwise trueness comparisons, all formulas differed significantly except for BU II versus Kane (p = 0.956) (Fig 2A). The precision (trimmed mean of SEQ-PE distances) ranged from 0.372 to 0.413 D among all formulas (Fig 1B). Pairwise analysis showed significant differences only between Kane versus Hoffer QST (0.01 ≤ p < 0.05), and between Kane versus Hill RBF 3.0 (0.01 ≤ p < 0.05) (Fig 2B). The trimmed mean absolute SEQ-PE (accuracy) ranged from 0.376 to 0.445 D across all formulas (Fig 1C). Kane showed a significantly lower trimmed-mean absolute SEQ-PE than Hoffer QST (0.376 vs. 0.445 D, p = 0.014), whereas no other pairwise accuracy comparisons reached statistical significance (Fig 2C). The trimmed-mean absolute SEQ-PE estimates and their 95% confidence intervals showed substantial overlap across formulas. Across all formulas, 30–39% of eyes were within ±0.25 D, 58–66% within ±0.50 D, 77–86% within ±0.75 D, and 89–95% within ±1.00 D of the predicted refraction (Fig 3). Cochran’s Q test revealed no statistically significant differences among formulas for any of the prediction error thresholds. In the MPE–adjusted sensitivity analysis, the range of MAE narrowed from 0.407–0.477 D before adjustment to 0.404–0.441 D after adjustment. Cooke K6 and Hoffer QST, which showed larger directional MPEs before adjustment, demonstrated improved adjusted MAEs (0.409 D and 0.441 D, respectively). The overall difference in MPE-adjusted APE remained significant (Friedman p = 0.003), with Kane showing lower adjusted APE than Hill-RBF 3.0 and Hoffer QST after Holm correction (S1 Table).

**Fig 1:**
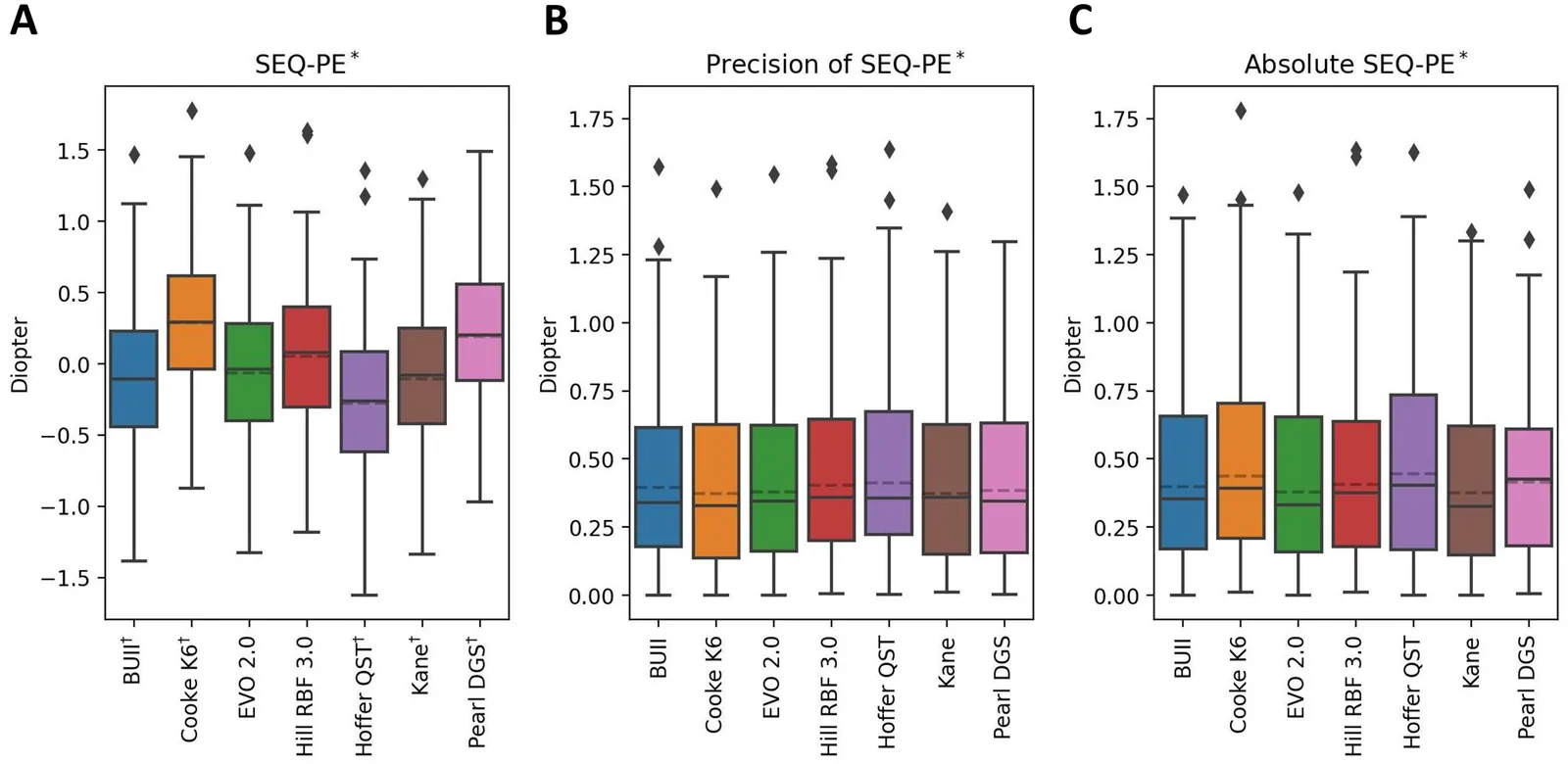
Box-plot graphs of (A) spherical equivalent prediction error (SEQ-PE; trueness), (B) precision, and (C) absolute spherical equivalent prediction error (absolute SEQ-PE; accuracy) for each intraocular lens power calculation formula. An asterisk (*) indicates significant differences between formulas, and a cross (†) indicates a trimmed mean SEQ-PE significantly different from zero.

**Fig 2:**
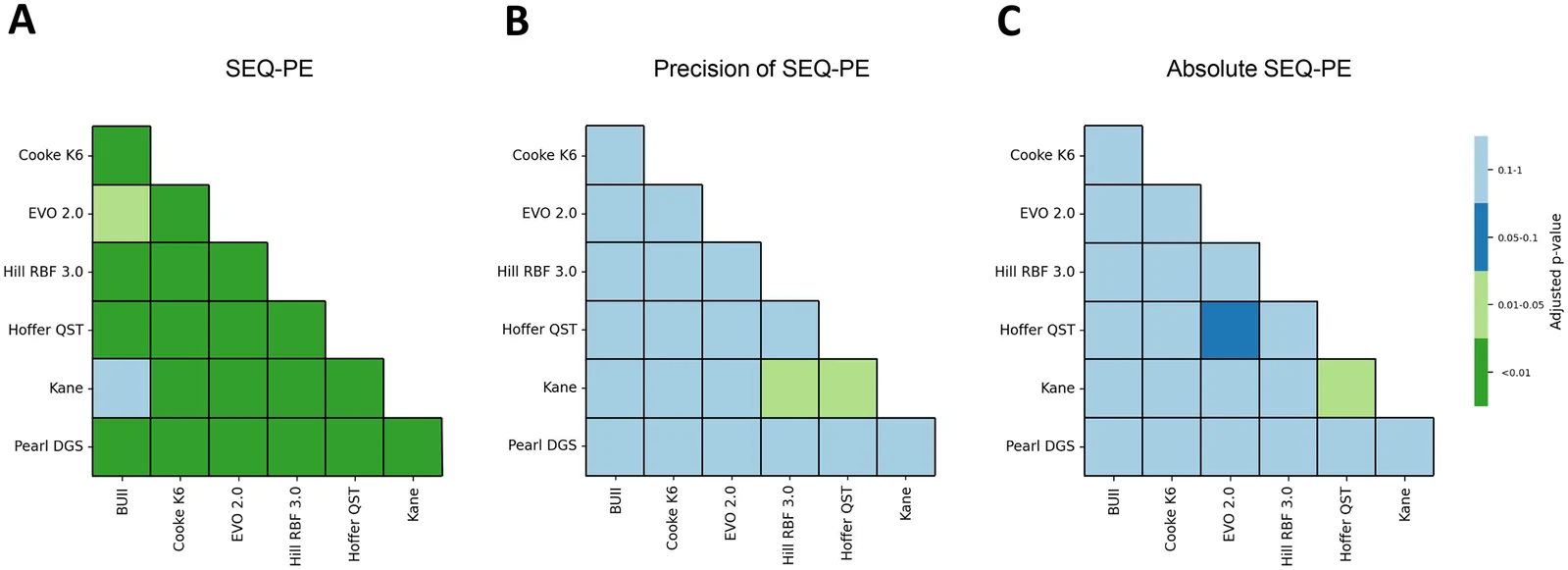
Heatmaps of pairwise comparisons for (A) trueness, (B) precision, and (C) accuracy among seven IOL power calculation formulas based on trimmed-mean analysis with Holm-adjusted p-values.

**Fig 3:**
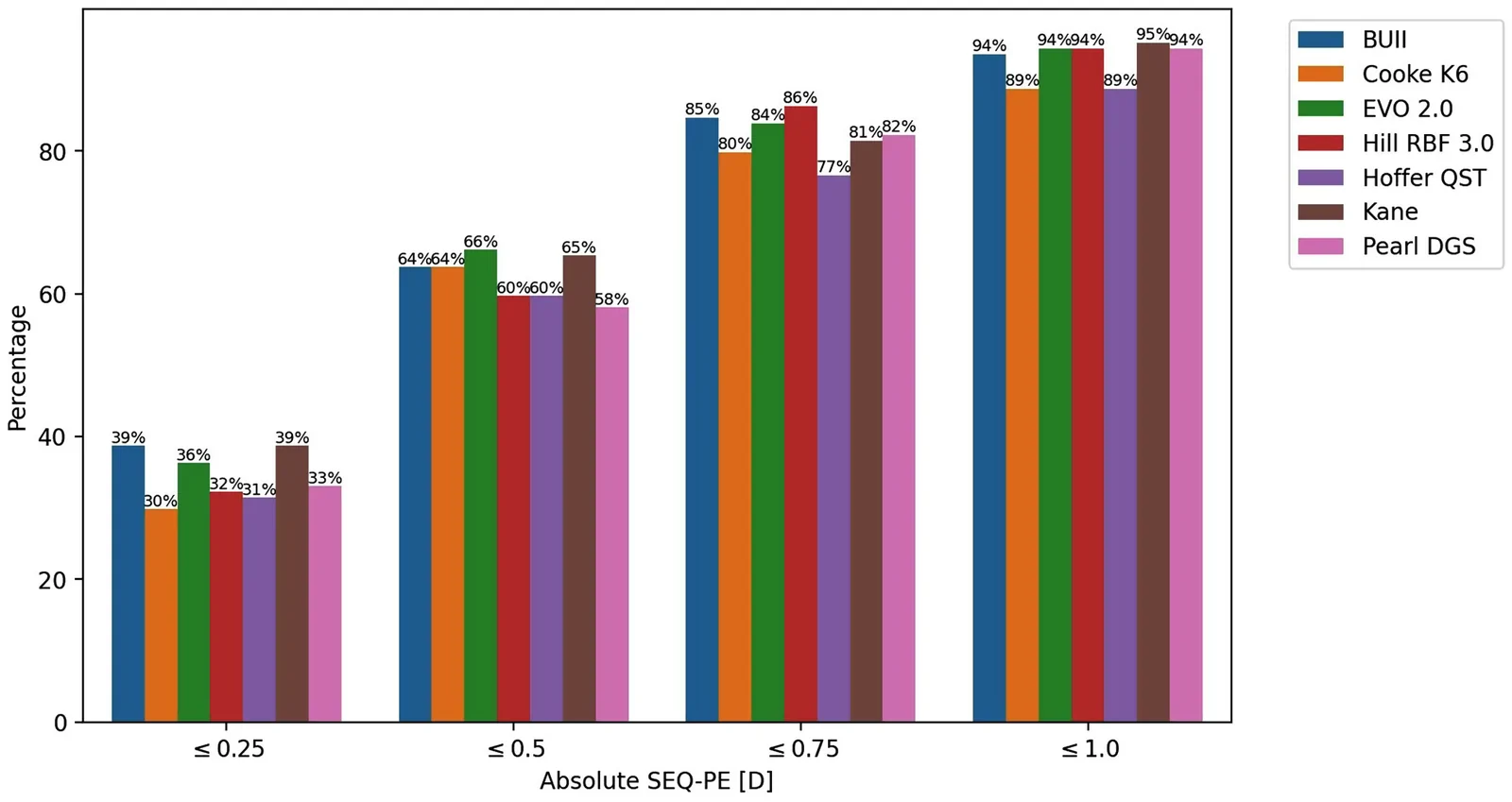
Ratios of eyes within absolute spherical equivalent prediction error (SEQ-PE) thresholds of 0.25, 0.50, 0.75 and 1.00 D for each formula.

Correlation analysis revealed that most formulas showed weak associations between ACD and PE (Fig 4). BU II, EVO 2.0, Hill RBF 3.0, Hoffer QST, Kane, and Pearl DGS did not demonstrate significant correlations with ACD (all p > 0.05). Only Cooke K6 showed a weak negative correlation with ACD (r = −0.18, p = 0.047), indicating a nominal tendency toward hyperopic PE in eyes with shallower anterior chambers. However, the magnitude of this correlation was small, indicating limited explanatory value. With regard to LT, Hoffer QST demonstrated a weak positive correlation between LT and PE (r = 0.23, p = 0.011), suggesting a gradual hyperopic shift with increasing LT (Fig 4). However, this association also explained only a small proportion of the variability in refractive outcomes and should not be interpreted as clinically actionable. EVO 2.0 (r = 0.16, p = 0.083) and Hill RBF 3.0 (r = 0.15, p = 0.097) showed borderline trends, whereas no significant correlations were observed for BU II, Cooke K6, Kane, or Pearl DGS. In the multivariable models with PE as the outcome, greater LT was associated with a more positive PE for Hill-RBF 3.0 (B = 0.514 D/mm, Holm-adjusted p = 0.046) and Hoffer QST (B = 0.634 D/mm, Holm-adjusted p = 0.005). However, neither ACD nor LT was independently associated with absolute PE after Holm correction, indicating that these associations reflected directional bias rather than a consistent increase in error magnitude (S2 Table).

**Fig 4:**
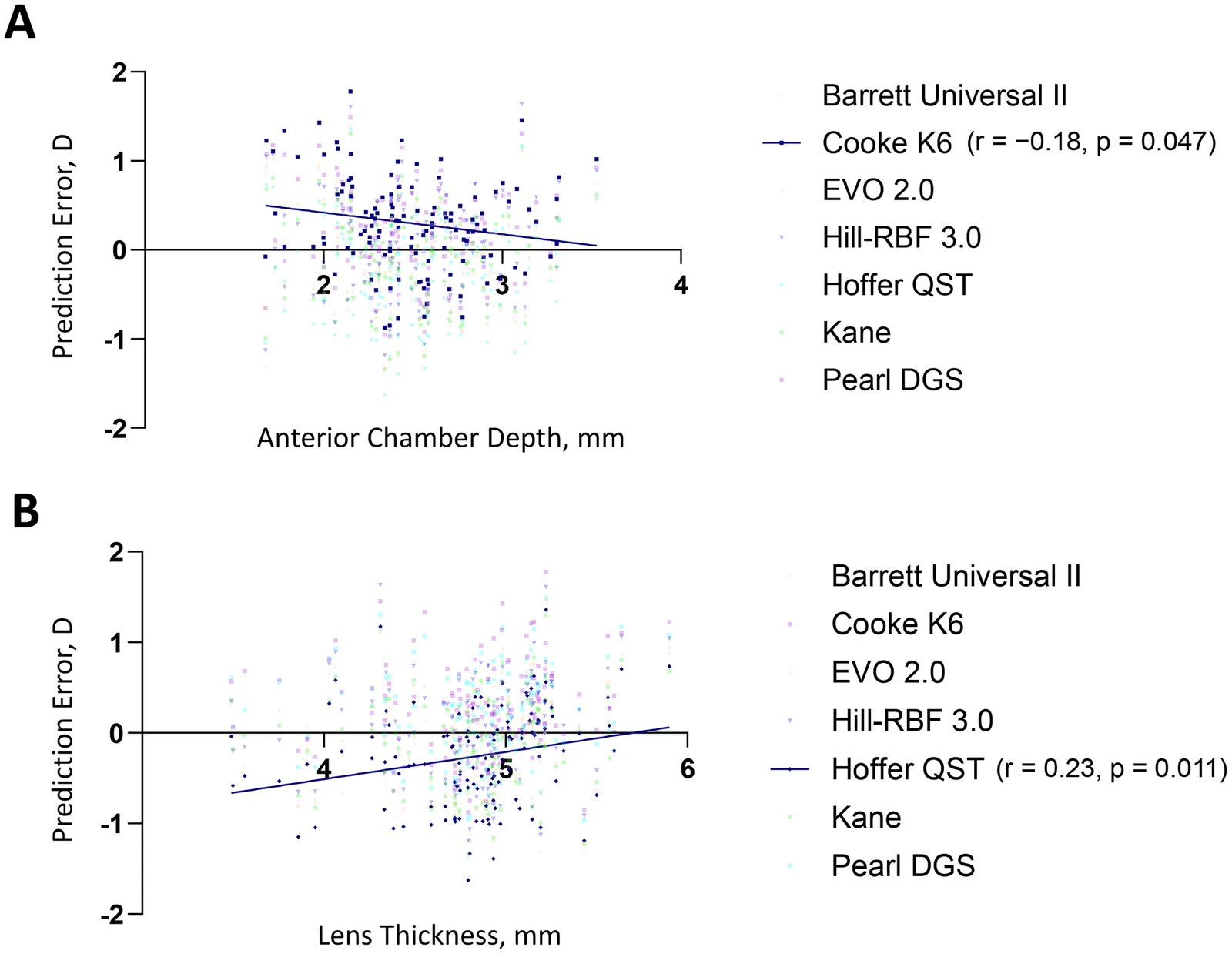
Scatter plots demonstrating the relationship between anterior chamber depth (ACD) and prediction error (PE) (A), and between lens thickness (LT) and prediction error (PE) (B) for the seven intraocular lens power calculation formulas.

When eyes were stratified by median ACD (2.44 mm), Cooke K6 demonstrated a significantly higher MAE in the shallow ACD group compared with the deeper ACD group (0.55 vs. 0.41 D, p = 0.030) (Table 3). When stratified by median LT (4.90 mm), Hoffer QST showed a slightly higher MAE in the thin lens group compared with the thick lens group, but the difference was not statistically significant (0.53 vs. 0.42 D, p = 0.092) (Table 3). The subgroup analyses were exploratory, and the nominal P values were not adjusted for multiple comparisons.

**Table 3:** Comparison of Mean Absolute Prediction Error According to Anterior Chamber Depth and Lens Thickness.

Formula	MAE (Low ACD), D	MAE (High ACD), D	P-value	MAE (Thin LT), D	MAE (Thick LT), D	P-value
Barrett Universal II	0.46	0.41	0.335	0.43	0.44	0.983
Cooke K6	0.55	0.41	**0.030**	0.44	0.51	0.279
EVO 2.0	0.44	0.38	0.316	0.39	0.43	0.540
Hill-RBF 3.0	0.45	0.43	0.685	0.41	0.47	0.329
Hoffer QST	0.50	0.46	0.542	0.53	0.42	0.092
Kane	0.43	0.38	0.366	0.39	0.42	0.615
Pearl DGS	0.47	0.41	0.319	0.43	0.46	0.634

ACD: anterior chamber depth; D: diopter; LT: lens thickness; MAE: mean absolute prediction error; MPE: mean prediction error.

Low and high ACD groups were defined based on the median ACD value (2.44 mm), and thin and thick LT groups were defined based on the median LT value (4.90 mm).

Values with statistical significance are shown in boldface.

## Discussion

In this study, we compared the predictive performance of seven modern IOL calculation formulas in eyes with short axial length using the ESCRS calculator and the Eyetemis analysis tool. Overall, small differences in Eyetemis accuracy were observed among the formulas, with Kane showing the lowest and Hoffer QST the highest trimmed-mean absolute PE. However, no formula showed statistically significant superiority over the others with regard to the proportion of eyes within commonly accepted prediction error thresholds.

Kane showed the lowest trimmed-mean absolute PE in the Eyetemis analysis, but the magnitude of the differences was small and no significant inter-formula difference was observed at clinically relevant prediction-error thresholds. Therefore, the apparent numerical advantage should be interpreted cautiously, and the present data do not establish clear clinical superiority of Kane over other formulas. Kane integrates theoretical optics with artificial intelligence [12], while EVO 2.0 employs a thick-lens model incorporating posterior corneal curvature estimation [14]. Other contemporary formulas also differ substantially in their use of biometric and data-driven components. Barrett Universal II uses a theoretical model incorporating multiple biometric parameters; [10] Hill-RBF and Pearl-DGS incorporate artificial intelligence or machine-learning approaches; [11,13] and formulas such as Holladay 2 and Olsen incorporate additional anterior segment or patient-level variables relevant to ELP estimation [8,9]. Because these formulas model biometric relationships in nonlinear and formula-specific ways, simple univariable correlations between ACD or LT and prediction error may not fully capture the influence of anterior segment anatomy on formula performance.

Although the original Hoffer Q has historically been recommended for short eyes, it was not evaluated in the present study. Hoffer QST is a distinct formula and should not be interpreted as a direct surrogate for the original Hoffer Q. In our cohort, Hoffer QST demonstrated higher mean absolute error and lower precision compared with several newer formulas. Notably, Hoffer QST also showed a relatively large myopic MPE under the default setting, suggesting that its higher absolute error may have been partly attributable to systematic bias rather than the intrinsic performance of the formula. This interpretation was supported by the reduction in its MAE after formula-specific MPE adjustment. Nevertheless, prior studies have reported heterogeneous results. Gao et al. demonstrated superior performance of the Kane formula in short eyes and suggested that Pearl-DGS performed better in eyes with shorter axial length and deeper anterior chamber depth [23]. In contrast, Ma et al. reported higher accuracy of Barrett Universal II compared with Kane and EVO 2.0 in a Chinese population [24]. These discrepancies likely reflect population-specific biometric differences, highlighting the importance of external validation across diverse ethnic and anatomical cohorts.

Despite the numerically low absolute errors observed for Kane and EVO 2.0, no significant differences were detected among formulas when assessed by the proportions of eyes within ±0.25, ± 0.50, ± 0.75, or ±1.00 diopters. This suggests that although modern formulas differ in absolute error metrics, the clinical impact of these differences may be modest when high-quality optical biometry and default constants are used. From a practical standpoint, this finding highlights that modern formulas generally provide acceptable refractive accuracy in short eyes, rather than indicating a single definitively superior option.

In addition to overall formula performance, we further investigated the influence of ACD and LT on PE. For most formulas, ACD and LT were not consistently associated with absolute PE. However, the absence of statistically significant associations should not be interpreted as definitive evidence of biometric robustness or invariance. Cooke K6 exhibited higher absolute prediction error in eyes with shallower anterior chambers, whereas Hoffer QST also showed a tendency toward higher error in eyes with thinner crystalline lenses, although this subgroup difference was not statistically significant. In the multivariable analyses, greater LT was associated with a more positive directional PE for Hill-RBF 3.0 and Hoffer QST, whereas neither ACD nor LT was associated with absolute PE after Holm correction. This suggests a small formula-specific shift in the direction of prediction error rather than a consistent deterioration in overall accuracy. These findings suggest that certain formulas may show formula-specific associations with anterior segment geometry and lens-related factors in short eyes. However, because the correlation coefficients were small, these associations should be interpreted as exploratory and hypothesis-generating rather than as evidence that ACD or LT can be used to guide clinical formula selection. Subgroup analyses based on median stratification were performed for exploratory purposes, and we acknowledge that dichotomization may reduce statistical power. Multivariable regression analyses support a cautious interpretation of the median-split results and suggest that ACD and LT alone do not consistently explain formula performance in short eyes.

A major strength of this study is the combined use of the ESCRS calculator and the Eyetemis analysis platform. This approach differs from prior short-eye formula comparison studies by evaluating selected contemporary formulas within a standardized web-based calculator workflow and by separating systematic bias, precision, and absolute error using an Eyetemis-based analytic framework. The ESCRS calculator enables standardized comparison of multiple contemporary formulas under uniform biometric conditions, while Eyetemis provides ISO-compliant evaluation of trueness, precision, and accuracy using robust trimmed-mean statistical methods [18–20]. This distinction is clinically relevant because a formula with a low MAE may still have directional bias, whereas a formula with minimal bias may differ in precision. The present workflow therefore helps characterize formula performance more transparently than conventional MAE-based comparisons alone.

Several limitations should be acknowledged. First, the retrospective design may introduce selection bias. Second, although the sample size was sufficient for the primary comparison, it remained relatively modest for comparing seven formulas across multiple endpoints and for subgroup analyses stratified by ACD and LT. Therefore, the subgroup findings should be interpreted cautiously. Third, all patients received a single IOL model, limiting generalizability to other lens designs. Fourth, the cohort was predominantly female, consistent with the higher prevalence of short axial length in women. This imbalance may reflect smaller ocular dimensions in women and demographic features of the Korean cataract population, including age-related axial length shortening and longer female life expectancy [25]. Nevertheless, this sex imbalance limits the generalizability of the findings to male patients and to populations with different demographic or biometric characteristics. Fifth, postoperative refraction was assessed at 1 month. Because refractive stability may vary, particularly in anatomically short eyes, the use of a 1-month endpoint should be considered a limitation. Finally, institution-specific constant optimization was not performed, which may have influenced formula performance and warrants investigation in future studies. This limitation is particularly relevant for formulas showing relatively large MPEs, such as Cooke K6 and Hoffer QST, because their performance may have been affected by constant-related systematic bias. Another limitation is that the analysis was limited to selected ESCRS calculator formulas and did not include traditional benchmark formulas for short eyes, such as Hoffer Q.

In eyes with short axial length, modern IOL power calculation formulas demonstrated broadly comparable refractive accuracy with only small formula-specific differences. Although differences among formulas were small within clinically relevant prediction error ranges, anterior segment biometry was associated with subtle, formula-specific variations. Overall, these formulas showed broadly comparable refractive outcomes within the ESCRS calculator workflow, with only modest formula-specific effects of anterior segment biometry under default-constant conditions.

## Supporting information

S1 Table MPE-adjusted sensitivity analysis. (DOCX)

S2 Table Multivariable regression analyses of prediction error and absolute prediction error. (DOCX)
