# Supplementary material for: Comparison of IOL power calculation formulas in short eyes using ESCRS calculator and eyetemis: Influence of anterior segment biometry
Source: PLoS One. 2026 Oct 27;21(10):e0361501. doi: 10.1371/journal.pone.0361501 (PMC13712612; doi:10.1371/journal.pone.0361501)
Supplement: Supplementary file 1 [file pone.0361501.s001.docx]

**S1 Table. MPE-adjusted sensitivity analysis.**

| **Formula** | **Original MPE** | **Original MAE** | **Original MedAE** | **MPE-adjusted MPE** | **MPE-adjusted MAE** | **MPE-adjusted MedAE** | **Adj within ±0.25 D (%)** | **Adj within ±0.50 D (%)** | **Adj within ±0.75 D (%)** | **Adj within ±1.00 D (%)** | **Adj within ±1.50 D (%)** |
| --- | --- | --- | --- | --- | --- | --- | --- | --- | --- | --- | --- |
| Barrett Universal II | -0.107 | 0.435 | 0.353 | 0.000 | 0.430 | 0.342 | 38.71 | 61.29 | 83.87 | 94.35 | 99.19 |
| Cooke K6 | 0.294 | 0.477 | 0.393 | 0.000 | 0.409 | 0.328 | 39.52 | 66.13 | 84.68 | 92.74 | 100.00 |
| EVO 2.0 | -0.064 | 0.413 | 0.330 | 0.000 | 0.411 | 0.344 | 39.52 | 65.32 | 87.90 | 92.74 | 99.19 |
| Hill-RBF 3.0 | 0.053 | 0.441 | 0.375 | 0.000 | 0.436 | 0.360 | 34.68 | 62.10 | 87.10 | 94.35 | 98.39 |
| Hoffer QST | -0.270 | 0.477 | 0.403 | 0.000 | 0.441 | 0.355 | 33.06 | 60.48 | 85.48 | 95.16 | 99.19 |
| Kane | -0.109 | 0.407 | 0.325 | 0.000 | 0.404 | 0.359 | 39.52 | 68.55 | 88.71 | 95.16 | 100.00 |
| Pearl DGS | 0.192 | 0.443 | 0.425 | 0.000 | 0.412 | 0.346 | 35.48 | 66.13 | 86.29 | 95.97 | 100.00 |
| Overall Friedman test for adjusted APE: chi-square=19.475, p=0.0034 | | | |  |  |  |  |  |  |  |  |
|  |  |  |  |  |  |  |  |  |  |  |  |
| **Formula 1** | **Formula 2** | **Mean difference in adjusted APE (F1-F2), D** | **Wilcoxon p** | **Holm-adjusted p** |  |  |  |  |  |  |  |
| Barrett Universal II | Cooke K6 | 0.0214 | 0.1058 | 1.0000 |  |  |  |  |  |  |  |
| Barrett Universal II | EVO 2.0 | 0.0191 | 0.1412 | 1.0000 |  |  |  |  |  |  |  |
| Barrett Universal II | Hill-RBF 3.0 | -0.0060 | 0.5962 | 1.0000 |  |  |  |  |  |  |  |
| Barrett Universal II | Hoffer QST | -0.0107 | 0.1366 | 1.0000 |  |  |  |  |  |  |  |
| Barrett Universal II | Kane | 0.0269 | 0.0143 | 0.2580 |  |  |  |  |  |  |  |
| Barrett Universal II | Pearl DGS | 0.0184 | 0.1502 | 1.0000 |  |  |  |  |  |  |  |
| Cooke K6 | EVO 2.0 | -0.0023 | 0.8050 | 1.0000 |  |  |  |  |  |  |  |
| Cooke K6 | Hill-RBF 3.0 | -0.0274 | 0.1019 | 1.0000 |  |  |  |  |  |  |  |
| Cooke K6 | Hoffer QST | -0.0320 | 0.0206 | 0.3501 |  |  |  |  |  |  |  |
| Cooke K6 | Kane | 0.0055 | 0.8468 | 1.0000 |  |  |  |  |  |  |  |
| Cooke K6 | Pearl DGS | -0.0029 | 0.6662 | 1.0000 |  |  |  |  |  |  |  |
| EVO 2.0 | Hill-RBF 3.0 | -0.0251 | 0.0691 | 0.9671 |  |  |  |  |  |  |  |
| EVO 2.0 | Hoffer QST | -0.0297 | 0.0086 | 0.1642 |  |  |  |  |  |  |  |
| EVO 2.0 | Kane | 0.0078 | 0.2886 | 1.0000 |  |  |  |  |  |  |  |
| EVO 2.0 | Pearl DGS | -0.0006 | 0.9811 | 1.0000 |  |  |  |  |  |  |  |
| Hill-RBF 3.0 | Hoffer QST | -0.0047 | 0.3979 | 1.0000 |  |  |  |  |  |  |  |
| Hill-RBF 3.0 | Kane | 0.0329 | 0.0019 | 0.0389 |  |  |  |  |  |  |  |
| Hill-RBF 3.0 | Pearl DGS | 0.0244 | 0.0599 | 0.8985 |  |  |  |  |  |  |  |
| Hoffer QST | Kane | 0.0375 | 0.0008 | 0.0177 |  |  |  |  |  |  |  |
| Hoffer QST | Pearl DGS | 0.0291 | 0.0379 | 0.6063 |  |  |  |  |  |  |  |
| Kane | Pearl DGS | -0.0084 | 0.4612 | 1.0000 |  |  |  |  |  |  |  |

MPE adjustment: adjusted PE = original PE - formula-specific MPE; adjusted APE = absolute (adjusted PE).
