# Supplementary material for: Comparison of IOL power calculation formulas in short eyes using ESCRS calculator and eyetemis: Influence of anterior segment biometry
Source: PLoS One. 2026 Oct 27;21(10):e0361501. doi: 10.1371/journal.pone.0361501 (PMC13712612; doi:10.1371/journal.pone.0361501)
Supplement: Supplementary file 2 [file pone.0361501.s002.docx]

**S2 Table. Multivariable regression analyses of prediction error and absolute prediction error.**

1. Full multivariable models with prediction error (PE) as the outcome

| **Formula** | **Covariate** | **B** | **SE** | **95% CI** | **P value** | **Holm-adjusted P*** | **Adjusted R²** | **N** |
| --- | --- | --- | --- | --- | --- | --- | --- | --- |
| Barrett Universal II | ACD | 0.2110 | 0.1914 | -0.1680 to 0.5901 | 0.2724 | 1.0000 | 0.0167 | 124 |
|  | LT | 0.3603 | 0.1760 | 0.0118 to 0.7088 | 0.0429 | 0.4287 |  |  |
|  | AL | 0.0560 | 0.4156 | -0.7670 to 0.8791 | 0.8930 | — |  |  |
|  | Mean K | 0.0778 | 0.1131 | -0.1462 to 0.3019 | 0.4929 | — |  |  |
|  | IOL power | 0.0272 | 0.0911 | -0.1532 to 0.2076 | 0.7659 | — |  |  |
|  | Age | -0.0047 | 0.0057 | -0.0161 to 0.0067 | 0.4165 | — |  |  |
|  | Female sex | -0.3431 | 0.1829 | -0.7052 to 0.0191 | 0.0631 | — |  |  |
| Cooke K6 | ACD | -0.1932 | 0.1824 | -0.5545 to 0.1681 | 0.2918 | 1.0000 | 0.0543 | 124 |
|  | LT | -0.0242 | 0.1677 | -0.3565 to 0.3080 | 0.8855 | 1.0000 |  |  |
|  | AL | -0.7366 | 0.3961 | -1.5212 to 0.0480 | 0.0655 | — |  |  |
|  | Mean K | -0.1282 | 0.1078 | -0.3417 to 0.0854 | 0.2371 | — |  |  |
|  | IOL power | -0.0682 | 0.0868 | -0.2402 to 0.1037 | 0.4336 | — |  |  |
|  | Age | 0.0005 | 0.0055 | -0.0104 to 0.0113 | 0.9316 | — |  |  |
|  | Female sex | -0.2527 | 0.1743 | -0.5979 to 0.0926 | 0.1499 | — |  |  |
| EVO 2.0 | ACD | -0.0929 | 0.1855 | -0.4603 to 0.2744 | 0.6174 | 1.0000 | 0.0145 | 124 |
|  | LT | 0.1842 | 0.1705 | -0.1536 to 0.5220 | 0.2824 | 1.0000 |  |  |
|  | AL | -0.4060 | 0.4028 | -1.2037 to 0.3917 | 0.3155 | — |  |  |
|  | Mean K | -0.0264 | 0.1096 | -0.2436 to 0.1907 | 0.8099 | — |  |  |
|  | IOL power | -0.0584 | 0.0883 | -0.2333 to 0.1164 | 0.5093 | — |  |  |
|  | Age | -0.0024 | 0.0056 | -0.0134 to 0.0086 | 0.6705 | — |  |  |
|  | Female sex | -0.3063 | 0.1772 | -0.6574 to 0.0447 | 0.0866 | — |  |  |
| Hill-RBF 3.0 | ACD | 0.4663 | 0.1875 | 0.0950 to 0.8376 | 0.0143 | 0.1571 | 0.0690 | 124 |
|  | LT | 0.5138 | 0.1724 | 0.1724 to 0.8553 | 0.0035 | 0.0455 |  |  |
|  | AL | -0.1797 | 0.4071 | -0.9860 to 0.6266 | 0.6597 | — |  |  |
|  | Mean K | 0.0693 | 0.1108 | -0.1502 to 0.2888 | 0.5328 | — |  |  |
|  | IOL power | 0.0223 | 0.0892 | -0.1544 to 0.1990 | 0.8028 | — |  |  |
|  | Age | -0.0019 | 0.0056 | -0.0130 to 0.0093 | 0.7424 | — |  |  |
|  | Female sex | -0.2348 | 0.1791 | -0.5896 to 0.1200 | 0.1925 | — |  |  |
| Hoffer QST | ACD | 0.5025 | 0.1866 | 0.1328 to 0.8721 | 0.0081 | 0.0978 | 0.0798 | 124 |
|  | LT | 0.6336 | 0.1716 | 0.2937 to 0.9735 | 0.0003 | 0.0048 |  |  |
|  | AL | -0.3279 | 0.4053 | -1.1306 to 0.4749 | 0.4202 | — |  |  |
|  | Mean K | -0.0310 | 0.1103 | -0.2495 to 0.1875 | 0.7793 | — |  |  |
|  | IOL power | -0.0220 | 0.0888 | -0.1979 to 0.1540 | 0.8049 | — |  |  |
|  | Age | -0.0027 | 0.0056 | -0.0138 to 0.0084 | 0.6257 | — |  |  |
|  | Female sex | -0.2349 | 0.1783 | -0.5881 to 0.1183 | 0.1904 | — |  |  |
| Kane | ACD | 0.1193 | 0.1830 | -0.2431 to 0.4817 | 0.5157 | 1.0000 | -0.0161 | 124 |
|  | LT | 0.1758 | 0.1683 | -0.1575 to 0.5090 | 0.2983 | 1.0000 |  |  |
|  | AL | -0.4554 | 0.3973 | -1.2424 to 0.3316 | 0.2541 | — |  |  |
|  | Mean K | -0.0311 | 0.1082 | -0.2453 to 0.1832 | 0.7745 | — |  |  |
|  | IOL power | -0.0566 | 0.0871 | -0.2291 to 0.1159 | 0.5173 | — |  |  |
|  | Age | -0.0003 | 0.0055 | -0.0112 to 0.0106 | 0.9544 | — |  |  |
|  | Female sex | -0.1403 | 0.1748 | -0.4866 to 0.2060 | 0.4238 | — |  |  |
| Pearl DGS | ACD | -0.0948 | 0.1840 | -0.4591 to 0.2696 | 0.6074 | 1.0000 | -0.0017 | 124 |
|  | LT | 0.0207 | 0.1692 | -0.3144 to 0.3557 | 0.9030 | 1.0000 |  |  |
|  | AL | -0.1634 | 0.3995 | -0.9546 to 0.6278 | 0.6833 | — |  |  |
|  | Mean K | 0.0622 | 0.1087 | -0.1531 to 0.2776 | 0.5682 | — |  |  |
|  | IOL power | 0.0148 | 0.0876 | -0.1586 to 0.1882 | 0.8663 | — |  |  |
|  | Age | 0.0008 | 0.0055 | -0.0102 to 0.0117 | 0.8902 | — |  |  |
|  | Female sex | -0.2871 | 0.1758 | -0.6353 to 0.0610 | 0.1051 | — |  |  |

2. Full multivariable models with absolute prediction error (absolute PE) as the outcome

| **Formula** | **Covariate** | **B** | **SE** | **95% CI** | **P value** | **Holm-adjusted P*** | **Adjusted R²** | **N** |
| --- | --- | --- | --- | --- | --- | --- | --- | --- |
| Barrett Universal II | ACD | -0.0369 | 0.1192 | -0.2729 to 0.1992 | 0.7577 | 1.0000 | 0.0022 | 124 |
|  | LT | 0.0342 | 0.1096 | -0.1829 to 0.2512 | 0.7556 | 1.0000 |  |  |
|  | AL | -0.0047 | 0.2588 | -0.5173 to 0.5079 | 0.9856 | — |  |  |
|  | Mean K | -0.0120 | 0.0705 | -0.1515 to 0.1276 | 0.8655 | — |  |  |
|  | IOL power | 0.0308 | 0.0567 | -0.0816 to 0.1431 | 0.5887 | — |  |  |
|  | Age | -0.0036 | 0.0036 | -0.0107 to 0.0035 | 0.3159 | — |  |  |
|  | Female sex | 0.0158 | 0.1139 | -0.2098 to 0.2413 | 0.8901 | — |  |  |
| Cooke K6 | ACD | -0.0960 | 0.1260 | -0.3455 to 0.1534 | 0.4473 | 1.0000 | 0.0607 | 124 |
|  | LT | 0.0213 | 0.1158 | -0.2081 to 0.2507 | 0.8543 | 1.0000 |  |  |
|  | AL | -0.0290 | 0.2735 | -0.5708 to 0.5127 | 0.9157 | — |  |  |
|  | Mean K | 0.0306 | 0.0745 | -0.1169 to 0.1781 | 0.6820 | — |  |  |
|  | IOL power | 0.0689 | 0.0600 | -0.0498 to 0.1877 | 0.2525 | — |  |  |
|  | Age | -0.0024 | 0.0038 | -0.0099 to 0.0051 | 0.5314 | — |  |  |
|  | Female sex | -0.0933 | 0.1204 | -0.3317 to 0.1451 | 0.4397 | — |  |  |
| EVO 2.0 | ACD | -0.0974 | 0.1145 | -0.3242 to 0.1293 | 0.3964 | 1.0000 | 0.0233 | 124 |
|  | LT | 0.0420 | 0.1053 | -0.1665 to 0.2505 | 0.6904 | 1.0000 |  |  |
|  | AL | 0.3837 | 0.2486 | -0.1087 to 0.8761 | 0.1255 | — |  |  |
|  | Mean K | 0.0968 | 0.0677 | -0.0372 to 0.2309 | 0.1551 | — |  |  |
|  | IOL power | 0.1045 | 0.0545 | -0.0034 to 0.2124 | 0.0576 | — |  |  |
|  | Age | -0.0054 | 0.0034 | -0.0122 to 0.0014 | 0.1215 | — |  |  |
|  | Female sex | 0.0248 | 0.1094 | -0.1919 to 0.2415 | 0.8211 | — |  |  |
| Hill-RBF 3.0 | ACD | 0.1047 | 0.1114 | -0.1159 to 0.3254 | 0.3491 | 1.0000 | 0.0474 | 124 |
|  | LT | 0.1969 | 0.1025 | -0.0060 to 0.3998 | 0.0571 | 0.7991 |  |  |
|  | AL | 0.1842 | 0.2419 | -0.2950 to 0.6634 | 0.4480 | — |  |  |
|  | Mean K | 0.0668 | 0.0659 | -0.0636 to 0.1973 | 0.3125 | — |  |  |
|  | IOL power | 0.0869 | 0.0530 | -0.0182 to 0.1919 | 0.1041 | — |  |  |
|  | Age | -0.0057 | 0.0033 | -0.0123 to 0.0009 | 0.0892 | — |  |  |
|  | Female sex | -0.0199 | 0.1065 | -0.2307 to 0.1910 | 0.8522 | — |  |  |
| Hoffer QST | ACD | -0.2290 | 0.1315 | -0.4894 to 0.0314 | 0.0842 | 1.0000 | 0.0332 | 124 |
|  | LT | -0.2011 | 0.1209 | -0.4406 to 0.0383 | 0.0988 | 1.0000 |  |  |
|  | AL | 0.3371 | 0.2855 | -0.2284 to 0.9025 | 0.2402 | — |  |  |
|  | Mean K | 0.0728 | 0.0777 | -0.0812 to 0.2267 | 0.3512 | — |  |  |
|  | IOL power | 0.0847 | 0.0626 | -0.0393 to 0.2086 | 0.1787 | — |  |  |
|  | Age | -0.0049 | 0.0039 | -0.0127 to 0.0029 | 0.2155 | — |  |  |
|  | Female sex | 0.1045 | 0.1256 | -0.1443 to 0.3533 | 0.4073 | — |  |  |
| Kane | ACD | -0.0423 | 0.1126 | -0.2653 to 0.1807 | 0.7078 | 1.0000 | 0.0298 | 124 |
|  | LT | 0.0558 | 0.1035 | -0.1492 to 0.2609 | 0.5907 | 1.0000 |  |  |
|  | AL | 0.3681 | 0.2445 | -0.1162 to 0.8524 | 0.1350 | — |  |  |
|  | Mean K | 0.0834 | 0.0666 | -0.0485 to 0.2152 | 0.2128 | — |  |  |
|  | IOL power | 0.1069 | 0.0536 | 0.0008 to 0.2131 | 0.0484 | — |  |  |
|  | Age | -0.0042 | 0.0034 | -0.0109 to 0.0025 | 0.2132 | — |  |  |
|  | Female sex | 0.0667 | 0.1076 | -0.1464 to 0.2798 | 0.5367 | — |  |  |
| Pearl DGS | ACD | -0.0688 | 0.1146 | -0.2958 to 0.1582 | 0.5493 | 1.0000 | 0.0046 | 124 |
|  | LT | 0.0346 | 0.1054 | -0.1742 to 0.2433 | 0.7435 | 1.0000 |  |  |
|  | AL | 0.1441 | 0.2489 | -0.3488 to 0.6371 | 0.5636 | — |  |  |
|  | Mean K | 0.0853 | 0.0678 | -0.0489 to 0.2195 | 0.2107 | — |  |  |
|  | IOL power | 0.0753 | 0.0546 | -0.0328 to 0.1833 | 0.1703 | — |  |  |
|  | Age | -0.0026 | 0.0034 | -0.0094 to 0.0042 | 0.4447 | — |  |  |
|  | Female sex | -0.0938 | 0.1095 | -0.3107 to 0.1232 | 0.3937 | — |  |  |

ACD, anterior chamber depth; AL, axial length; CI, confidence interval; IOL, intraocular lens; K, keratometry; LT, lens thickness; PE, prediction error; SE, standard error.

Regression coefficients are unstandardized coefficients (B). Separate multivariable models were fitted for each formula and outcome, with ACD, LT, AL, mean K, implanted IOL power, age, and sex included simultaneously as covariates.

*Holm adjustment was applied to the 14 ACD/LT coefficients (2 covariates × 7 formulas) separately for each outcome. Female sex was coded as 1 and male sex as 0. B represents the expected change in the outcome per one-unit increase in a continuous covariate or for female versus male sex, with other covariates held constant.
